# Risk of Injection-Site Abscess among Infants Receiving a Preservative-Free, Two-Dose Vial Formulation of Pneumococcal Conjugate Vaccine in Kenya

**DOI:** 10.1371/journal.pone.0141896

**Published:** 2015-10-28

**Authors:** Deron C. Burton, Godfrey M. Bigogo, Allan O. Audi, John Williamson, Kenneth Munge, Jackline Wafula, Dominic Ouma, Sammy Khagayi, Isaac Mugoya, James Mburu, Shadrack Muema, Evasius Bauni, Tahreni Bwanaali, Daniel R. Feikin, Peter M. Ochieng, Ondari D. Mogeni, George A. Otieno, Beatrice Olack, Tatu Kamau, Melissa K. Van Dyke, Robert Chen, Paddy Farrington, Joel M. Montgomery, Robert F. Breiman, J. Anthony G. Scott, Kayla F. Laserson

**Affiliations:** 1 Kenya Medical Research Institute (KEMRI)/Centers for Disease Control and Prevention (CDC) Research and Public Health Collaboration, Kisumu and Nairobi, Kenya; 2 International Emerging Infections Program, Global Disease Detection Response Center, CDC, Kisumu and Nairobi, Kenya; 3 KEMRI-Wellcome Trust Research Programme, Kilifi, Kenya; 4 Center for Global Health, CDC, Atlanta, Georgia, United States of America; 5 Division of Vaccines and Immunization, Ministry of Public Health and Sanitation, Nairobi, Kenya; 6 GlaxoSmithKline Vaccines, Wavre, Belgium; 7 National Center for HIV/AIDS, Viral Hepatitis, STD, and TB Prevention, CDC, Atlanta, Georgia, United States of America; 8 Open University, Buckinghamshire, United Kingdom; 9 London School of Hygiene & Tropical Medicine, London, United Kingdom; University of Otago, NEW ZEALAND

## Abstract

There is a theoretical risk of adverse events following immunization with a preservative-free, 2-dose vial formulation of 10-valent-pneumococcal conjugate vaccine (PCV10). We set out to measure this risk. Four population-based surveillance sites in Kenya (total annual birth cohort of 11,500 infants) were used to conduct a 2-year post-introduction vaccine safety study of PCV10. Injection-site abscesses occurring within 7 days following vaccine administration were clinically diagnosed in all study sites (passive facility-based surveillance) and, also, detected by caregiver-reported symptoms of swelling plus discharge in two sites (active household-based surveillance). Abscess risk was expressed as the number of abscesses per 100,000 injections and was compared for the second vs first vial dose of PCV10 and for PCV10 vs pentavalent vaccine (comparator). A total of 58,288 PCV10 injections were recorded, including 24,054 and 19,702 identified as first and second vial doses, respectively (14,532 unknown vial dose). The risk ratio for abscess following injection with the second (41 per 100,000) vs first (33 per 100,000) vial dose of PCV10 was 1.22 (95% confidence interval [CI] 0.37–4.06). The comparator vaccine was changed from a 2-dose to 10-dose presentation midway through the study. The matched odds ratios for abscess following PCV10 were 1.00 (95% CI 0.12–8.56) and 0.27 (95% CI 0.14–0.54) when compared to the 2-dose and 10-dose pentavalent vaccine presentations, respectively. In Kenya immunization with PCV10 was not associated with an increased risk of injection site abscess, providing confidence that the vaccine may be safely used in Africa. The relatively higher risk of abscess following the 10-dose presentation of pentavalent vaccine merits further study.

## Introduction

Globally, *Streptococcus pneumoniae* causes an estimated 14.5 million episodes of serious disease (including pneumonia, meningitis, and sepsis) and over 800,000 deaths annually in children aged <5 years, with most pneumococcal-associated serious morbidity and mortality occurring in low- and middle-income countries [1]. Since 2006, the World Health Organization (WHO) has recommended that pneumococcal conjugate vaccine (PCV) be included in all national immunization programs, particularly in countries with high child mortality (i.e., under 5 mortality rate of >50 deaths/1,000 births) [2–3]. The Kenya Ministry of Public Health and Sanitation (MoPHS) applied for and received financial support from the GAVI Alliance to introduce a 10-valent formulation of PCV (PCV10) manufactured by GlaxoSmithKline Vaccines (GSK).

In March 2009, GSK presented an application to the WHO prequalification committee for a 2-aliquot, preservative-free presentation of PCV10. (To avoid ambiguity, we hereafter refer to the sequence of immunizations with the same antigen received by the same child as the “dose” number and the sequence of volumes of vaccine withdrawn from a single vial as the “aliquot” number. Thus, each child could receive 3 doses of PCV10 and each vial of PCV10 contained two aliquots.) A preservative-free, multi-aliquot vial vaccine formulation had not previously been used in developing countries. Theoretically, this formulation could result in adverse events following immunization (AEFI) if the second aliquot became contaminated before use—for example, if the second aliquot of PCV10 was not used within 6 hours of opening the vial. Lacking data or experience from a developing country, the WHO pre-qualification committee requested that the manufacturer work with an early-introduction developing country to characterize the magnitude of AEFI risk associated with PCV10 following appropriate training of the health workers who administer vaccines.

In late 2009, GSK agreed to fund two longstanding research collaborations with the Kenya Medical Research Institute (KEMRI)—the KEMRI-Wellcome Trust Research Programme in Kilifi and the KEMRI/Centers for Disease Control and Prevention (CDC) Research and Public Health Collaboration in Nairobi and Kisumu—to conduct a two-year post-introduction, or Phase IV, vaccine safety study of PCV10 in existing population-based health and demographic surveillance platforms, referred to as the Vaccine Adverse Events in Kenya study (VAEIK). On the basis of the establishment of this prospective safety study, the WHO pre-qualification committee approved the use of PCV10 in Kenya. Here, we report on the risks of injection site abscess following immunization with second compared to first aliquots of PCV10 and with PCV10 compared to pentavalent vaccine (the comparator) for the two-year VAEIK study period, which concluded February 13, 2013.

## Materials and Methods

### Study Sites

**Fig 1:**
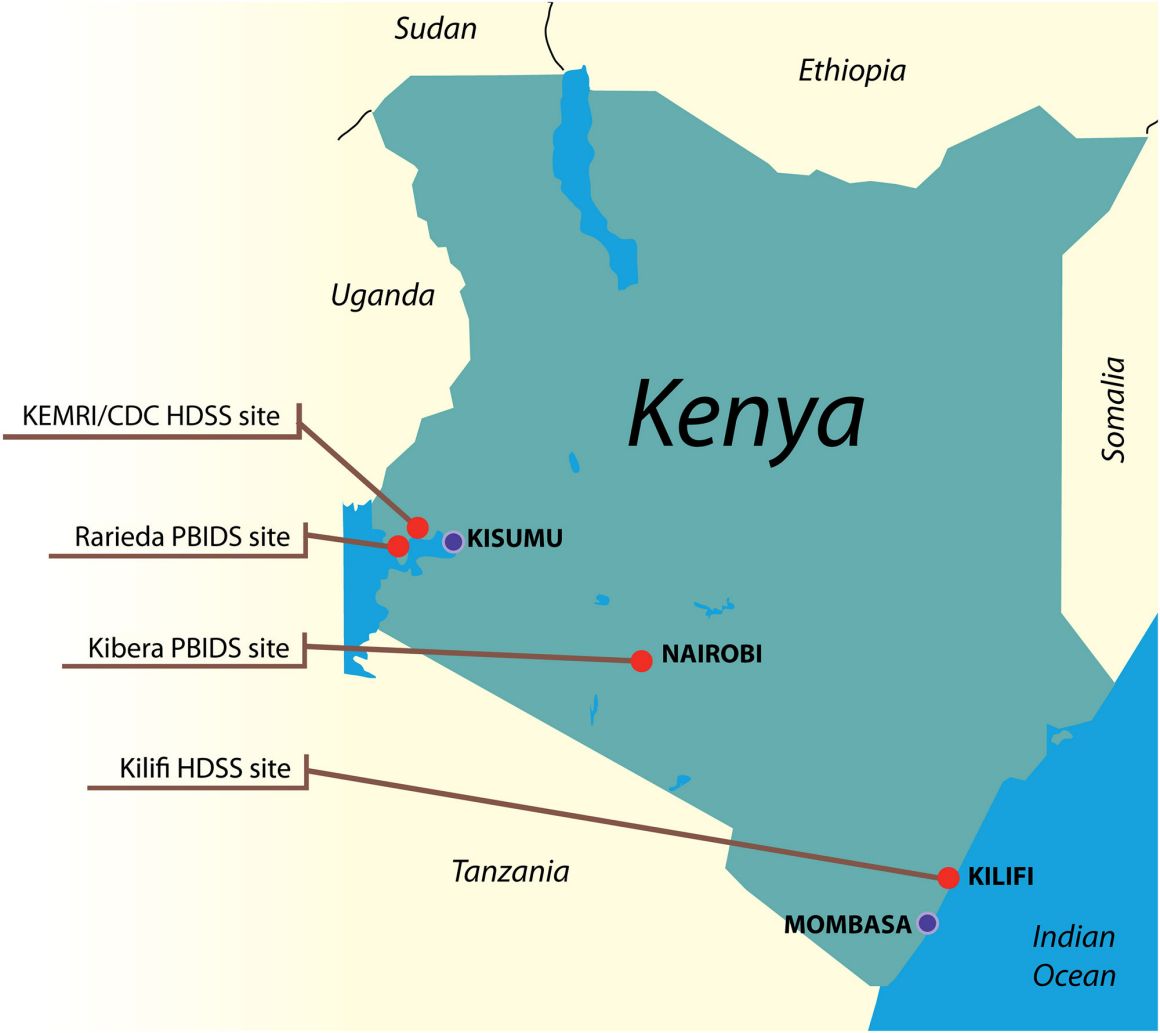
Vaccine Adverse Events in Kenya (VAEIK) study sites. Map of Kenya showing VAEIK study sites (red circles). Abbreviations: CDC, Centers for Disease Control and Prevention; HDSS, Health and Demographic Surveillance System; PBIDS, Population-based Infectious Disease Surveillance Site; KEMRI, Kenya Medical Research Institute. Reprinted from original artist under a CC BY license, with permission from Alan Rubin, original copyright 2013.

The VAEIK study is a Phase IV study conducted at four sites: one site operated by the KEMRI-Wellcome Trust Programme—the Kilifi Health and Demographic Surveillance System (HDSS) site at the Kenyan coast—and three sites operated by the KEMRI/CDC Research and Public Health Collaboration—the Kibera population-based infectious disease surveillance (PBIDS) site in Nairobi, the Rarieda PBIDS site in western Kenya, and a portion of the KEMRI/CDC HDSS site also in western Kenya and including parts of Rarieda and Siaya Districts (Fig 1). The Rarieda PBIDS site is geographically embedded within the KEMRI/CDC HDSS, but for purposes of this study, data reported for the KEMRI/CDC HDSS site do not include data from Rarieda PBIDS. The four study sites have a combined annual birth cohort of approximately 11,500 infants (Kilifi 9,000, Kibera 1,000, Rarieda 1,000, and the included portion of the KEMRI/CDC HDSS 500 infants).

### Kilifi HDSS site

The Kilifi HDSS has operated since the year 2000, and its population register is maintained by visits to each household every four months [4]. All children born to mothers resident in the Kilifi HDSS were included in the VAEIK study. In-migrating children aged <1 year also were included in the VAEIK study if they fulfilled the criteria for residence, defined as a person who intended to live at the address where he/she was found for at least 3 months, and who had already spent at least one night at this address. As of March 2011, the Kilifi HDSS had a resident population of 261,919, including 10,681 infants aged <1 year. The complete (3-dose) pentavalent vaccine coverage among children aged <1 year in the Kilifi HDSS was 91–96% [5] and the infant mortality ratio (IMR) was 24.4/1,000 live births.

### PBIDS sites in Kibera and Rarieda

Since 2005, KEMRI/CDC has operated PBIDS in a rural area of Rarieda District in western Kenya and in a western section of the Kibera informal settlement in Nairobi, as described previously [6–7]. In both sites, PBIDS enrolled residents who had lived in the catchment area for at least 4 months. To be enrolled in the Rarieda PBIDS site, adult participants also had to be registered with the KEMRI/CDC HDSS. All infants born to mothers enrolled as participants in PBIDS automatically were included in the VAEIK study unless the mothers requested otherwise. In 2011, the Kibera PBIDS site had approximately 27,800 participants including 723 infants aged <1 year; the infant mortality rate was 50/1,000 person-years [8]. The Rarieda PBIDS site had approximately 25,470 participants residing in 33 villages within Rarieda District in 2011, including 855 infants aged <1 year. In Rarieda in 2010, the complete pentavalent vaccine coverage of infants aged <1 year was 85% and the IMR was 53/1,000 live births.

### KEMRI/CDC HDSS site in western Kenya

Within a subset of the KEMRI/CDC HDSS in western Kenya, VAEIK study data were collected at two health facilities in Siaya County: Ting’wang’i Health Centre (entire VAEIK study period) and Njejra Health Centre (4 months, March-June 2011). The VAEIK study population included infants enrolled in the KEMRI/CDC HDSS and residing in 26 villages within a 5 km radius around Ting’wang’i Health Centre and 20 villages within a 3 km radius around Njejra Health Centre in Siaya County. The KEMRI/CDC HDSS enrolled residents who had lived in the catchment area for at least 4 months. Infants born to enrolled mothers automatically became HDSS participants and were eligible for inclusion in the VAEIK study unless their mothers requested otherwise. In 2011, the portion of the KEMRI/CDC HDSS included in the VAEIK study had a total resident population of 17,200 and an annual birth cohort of 500 infants. In the overall KEMRI/CDC HDSS in 2010, the complete pentavalent vaccine coverage of infants aged <1 year was 85% and the IMR was 67/1,000 live births.

### PCV10 Introduction in Kenya

PCV10 was officially introduced into the national immunization program in Kenya on February 14, 2011 with an active publicity campaign, and provided free of charge for infants country-wide. The vaccine booklet supplied by the Kenya MoPHS and training of immunization workers stipulated that PCV10 would be administered in the right thigh while pentavalent vaccine (which contains diphtheria and tetanus toxoids, whole cell pertussis vaccine, *H*. *influenzae* type b conjugate vaccine, and Hepatitis B vaccine delivered in a single needle) would be administered in the left thigh. The MoPHS policy indicated that each of these vaccines should be given simultaneously to infants 6, 10 and 14 weeks of age. In addition, the MoPHS offered catch-up vaccination, in 3 doses four weeks apart, to any child aged <12 months presenting to a vaccine clinic during 2011. The MoPHS provided centralized, in-person training on PCV10 immunization practices to vaccinators in each district and distributed written guidance on correct handling and administration of PCV10. The MoPHS guidance informed vaccinators that the PCV10 two-dose vial formulation lacked a preservative and instructed that opened vials must be discarded at the end of each immunization session, or after 6 hours from first opening, whichever came first. Vaccinators were not employed by the study projects at any of the VAEIK sites, and VAEIK study staff did not provide any training or information to immunization clinic personnel on infection control, appropriate vaccine handling or safe administration of vaccines, thus ensuring real world conditions for the safety of the immunizations.

### Inclusion Criteria

This report includes data from the entire two-year VAEIK study period: February 14, 2011 through February 13, 2013. In Kilifi, PCV10 was introduced on January 11, 2011, ahead of the official national launch; thus, Kilifi HDSS data also include the period January 11 through February 13, 2011. For a child to be included in the VAEIK study, he/she must have been aged <1 year and enrolled in HDSS or PBIDS surveillance in one of the study sites during the study period.

### Vaccine Surveillance and Aliquot Recording

#### Kilifi HDSS vaccine surveillance

In the Kilifi HDSS, a vaccine monitoring system existed in all health facilities. Vaccine clinic data from the health facilities were entered into computers directly at the point of immunization. The system captured information pertaining to childhood vaccines including all PCV10 doses given, date given and the number of the aliquot withdrawn from the vaccine vial, as recorded in the Mother Child Booklet by the vaccinator, and linked this directly to the population register of the Kilifi HDSS.

#### KEMRI/CDC HDSS vaccine surveillance

In the KEMRI/CDC HDSS site in western Kenya, vaccine clinic data were collected for purposes of the VAEIK study at Njejra and Ting’wang’i Health Centres. In addition, vaccination history was recorded for all HDSS children aged <2 years at household visits conducted every 4 months. At home visits, the preferred source of vaccine history data was the Mother Child Booklet, but verbal reports from caregivers also were accepted if the booklet was unavailable. Aliquot information for PCV10 was recorded in Mother Child Booklets by the vaccinators and abstracted by HDSS surveillance staff.

#### Rarieda & Kibera PBIDS vaccine surveillance

The Rarieda PBIDS site in western Kenya was embedded within the KEMRI/CDC HDSS, and vaccination history information collected during 4-monthly HDSS home visits were linked to PBIDS participants. In the Rarieda PBIDS site, vaccination data were obtained during immunization visits to the PBIDS study facility, Lwak Mission Hospital, and 5 other clinics. In addition, in both the Rarieda and Kibera PBIDS sites, routine household visits to collect morbidity data were conducted weekly (February through June 2011) or biweekly (beginning in July 2011) throughout the study period. At each home visit, community interviewers (CIs) recorded all vaccines received by children in the household since the previous home visit. As in the KEMRI/CDC HDSS, the preferred source of vaccine history data was the Mother Child Booklet, but verbal reports also were accepted if necessary. In the Kibera PBIDS site, vaccination data also were obtained during immunization visits to the PBIDS study facility, Tabitha Clinic. Vaccination data obtained during home and facility visits in the KEMRI/CDC HDSS and PBIDS sites included the date of vaccination, the dose number, the injection site, and the number of the aliquot withdrawn from the vaccine vial, as recorded in the Mother Child Booklet.

### Ascertainment of Injection Site Abscesses

Abscesses were detected through facility- and household-based surveillance in the two PBIDS sites, but only through facility-based surveillance in the two HDSS sites.

#### Facility-based abscess surveillance in Kilifi HDSS, KEMRI/CDC HDSS, and the two PBIDS sites

In all study sites, caregivers could bring a child with suspected AEFI, such as injection site abscess, back to the Maternal and Child Health (MCH) clinic or other vaccine delivery center where the vaccine was administered. All children aged <1 year with history of vaccination in the 7 days before the sick visit were assessed for possible AEFI if the presenting complaint included localized symptoms in an extremity (other than obvious trauma), and/or if the caregiver suspected the child had AEFI, and/or if the clinician suspected toxic shock syndrome. In all sites, a standardized form was used to capture demographic and clinical information, including vaccine history, reported local and systemic symptoms, physical examination findings (including detailed evaluation of injection site(s) for swelling [diameter was measured and recorded], induration, fluctuance, erythema, discharge, local hotness, blisters, red streaks, or swollen regional lymph nodes), circumference of right and left thighs (if there was history of thigh vaccination), interventions/treatments provided (such as surgical drainage, antibiotics, or anti-inflammatory/anti-pyretic medications), results of laboratory evaluations, if any, outcome of clinical encounter, and clinical impression, such as injection site abscess. Depending on symptom severity, MCH clinics could refer patients with suspected AEFI to a hospital for further evaluation.

In the Kilifi HDSS site, data were recorded on facility-based questionnaires by medical staff at any one of 26 health centers within the Kilifi HDSS. Data were checked on-site by the Kilifi VAEIK study project manager and scanned copies of completed facility-based questionnaires were stored. In study health facilities in the KEMRI/CDC HDSS (Njejra and Ting’wang’i Health Centres), Rarieda PBIDS (Lwak Mission Hospital) and Kibera PBIDS (Tabitha Clinic) surveillance sites, trained study clinicians entered data on suspected AEFI onto scannable paper forms.

#### Household-based abscess surveillance in the two PBIDS sites

Household-based detection of abscesses occurred in the Rarieda and Kibera PBIDS surveillance sites. Trained CIs visited the homes of all PBIDS participants weekly (through June 2011) or once every two weeks (beginning July 2011), and used personal digital assistants or smart phones to administer standard questions about vaccine history and morbidity [6]. If a caregiver reported that a child received an immunization since the last home visit, the caregiver was asked whether injection site inflammation was observed following the immunization. From February through June 2011, the caregiver was only asked to report injection site inflammation that occurred within 7 days after immunization, but beginning in July 2011 the caregiver was asked to report injection site inflammation occurring within 14 days after immunization and the date(s) when the injection site symptoms began. If injection site inflammation was reported, the caregiver was asked to report the presence or absence (and if present, the location and dates) of injection site swelling, redness, discharge, or other local symptoms (referred to collectively as “abscess related symptoms”). Information on systemic symptoms, such as subjective fever, were collected for all participants at every home visit. Children with reported injection site inflammation were referred to the local study health facility (Tabitha Clinic in Kibera and Lwak Mission Hospital in Rarieda District) for further evaluation.

#### Abscess definition, standardization and training

An injection site abscess is a localized soft tissue collection of material, occurring at the site of immunization [9]. For purposes of this study, two injection site abscess case definitions were used. First, in all of the study facilities, an injection site abscess was clinically diagnosed by the treating health care worker based on clinician’s judgment. Although laboratory tests such as full hemogram and blood cultures can be helpful to inform the management of an abscess, these were not frequently performed as part of routine care in the study areas; thus, laboratory criteria were not included in the case definition of an injection site abscess for this study. Rather, clinicians diagnosed injection site abscess using history and physical exam findings, in accordance with standardized training (described below). Second, for the household-based surveillance conducted in the two PBIDS sites, where no clinician was present, we used a modified version of a clinical case definition of injection site abscess from consensus guidelines published in 2007 by the Brighton Collaboration Local Reactions Working Group for Abscess at Injection Site [8], as follows: Mass or swelling at injection site AND spontaneous drainage of material.

Health workers (including clinical officers, medical officers and nurses) in all study sites received training on intramuscular abscess identification and management conducted by a pediatric surgeon from the University of Nairobi School of Medicine. In all study sites, health workers and data entry staff were trained on how to complete the VAEIK AEFI case report form used in the health facility-based surveillance. Monthly (KEMRI/CDC HDSS and PBIDS sites) or quarterly (Kilifi HDSS site) refresher trainings, specific to each site, were conducted, and in all sites, health workers were encouraged to complete both the Kenya MoPHS AEFI form and the VAEIK study case report form for all adverse events reported.

### Statistical Analysis

#### Analyses of main objectives

The primary objective was to evaluate whether the site of injection of a second aliquot of PCV10 was more likely to develop an abscess or related symptoms within 7 days than the site of injection of a first aliquot of PCV10. The 7-day time lag was chosen to optimize the specificity of the AEFI endpoint by restricting to events occurring within a short period of time following immunization. Specificity also was enhanced by limiting surveillance of abscess events to the appropriate thigh that received the vaccine injection. For the primary objective, we computed risk ratios (RRs) using log binomial regression. Ninety-five percent confidence intervals (CIs) and the corresponding p-values were calculated with generalized estimating equations using an exchangeable correlation structure to account for intra-child correlation in injection site abscesses or related symptoms. An additional objective was to evaluate whether an injection site in the leg that received PCV10 was more likely to develop an abscess or related symptoms within 7 days than an injection site in the leg that received pentavalent vaccine. The Mother Child Booklet contained pre-printed instructions directing vaccinators to place PCV10 only into the right thigh, and training provided by the MoPHS at the time of PCV10 introduction instructed vaccinators to place pentavalent vaccine only into the left thigh; for VAEIK analyses, correct vaccine placement was assumed. Exact conditional logistic regression was used to calculate matched odds ratios (mORs) and p-values to evaluate abscess risk among matched pairs of PCV10 and pentavalent vaccine injections received at the same immunization encounter; 95% CIs were obtained using the asymptotic method. Fisher’s exact test was used for risk comparisons with small cell sizes (<5). All analyses were performed in SAS version 9.2 (SAS Institute, Cary, North Carolina, USA).

#### Analyses related to different pentavalent vaccine formulations

Pentavalent vaccine was selected as the comparator because it was given according to the same 3-dose schedule as PCV10 and both vaccines were administered in a thigh. In addition, the GSK pentavalent vaccine Tritanrix™-HepB/Hiberix™ (presented as a 2-aliquot vial with the preservative thimerosal, and consisting of a liquid and freeze dried component which are mixed immediately prior to immunization) already was established in Kenya’s routine immunization program at the time of PCV10 introduction and was considered to have an acceptable safety profile. However, in July 2011, the Kenya Expanded Programme on Immunization initiated a switch from the GSK pentavalent vaccine to a Serum Institute of India (SII) pentavalent vaccine product (presented as a 10-aliquot vial with thimerosal). Within 3 months of introduction, all pentavalent immunizations delivered through Kenya’s routine immunization program used the new SII formulation.

Following the introduction of the new presentation of pentavalent vaccine, we noticed an increase in the frequency of reporting of abscesses in the control group and decided therefore to do an *ad hoc* analysis to compare observed risks of abscess or related symptoms between the two pentavalent vaccine formulations using data from the Kilifi HDSS, Rarieda PBIDS, and KEMRI/CDC HDSS sites. Data from the Kibera PBIDS site were not included in this analysis because Kibera PBIDS records did not distinguish whether observed abscesses or related symptoms occurred at a PCV10 injection site or a pentavalent vaccine injection site from February 2011 through July 2011 (i.e. whether the abscess was located on the left or right leg), limiting data available for analysis from the GSK pentavalent vaccine period. We also compared risks of abscess and related symptoms for PCV10 separately to each pentavalent vaccine formulation.

### Ethics Statement

The VAEIK protocol was reviewed and approved by KEMRI’s Ethical Review Committee (KEMRI #1873) and the Oxford Tropical Research Ethics Committee (#48–10). CDC’s Institutional Review Board approved the VAEIK protocol as non-research; the home-visit assessment of abscesses in Kibera and Rarieda was approved within an amendment to the PBIDS protocol (KEMRI #1899, CDC protocol #4566). Data collected in the KEMRI/CDC HDSS were approved through the HDSS protocol (KEMRI #1801, CDC #3308). Parents or guardians of all participants provided written informed consent to participate in surveillance of abscess-related health outcomes following immunization, as approved under the respective site protocols (KEMRI #1899/CDC #4566 for Kibera and Rarieda PBIDS sites; KEMRI #1801/CDC #3308 for KEMRI/CDC HDSS site; KEMRI #1873 for Kilifi HDSS site). Written informed consent for recording of vaccination history also was provided by parents or guardians in the KEMRI/CDC HDSS site and both PBIDS sites under the same approved protocols used for abscess surveillance. In the Kilifi HDSS, vaccine history monitoring was conducted by staff of the Kenya MOPHS under the Pneumococcal Conjugate Vaccine Impact Study (KEMRI #1433); the KEMRI Ethical Review Committee determined this vaccine history monitoring to be public health surveillance which did not require individual informed consent.

## Results

### Vaccine Dose and Aliquot Counts

A total of 58,288 doses of PCV10 and 46,265 doses of pentavalent vaccine were administered to enrolled infants at all four sites during the VAEIK study period (Table 1). The largest share of PCV10 (87.5%) and pentavalent vaccine (86.6%) doses were administered to children from the Kilifi HDSS. Overall, aliquot information was available for 43,756 (75.1%) PCV10 injections, and of these 24,054 (55.0%) were first aliquots and 19,702 (45.0%) were second aliquots (Table 2). The completeness of aliquot recording improved substantially from the first year (67.3%) to the second year (98.4%) of the VAEIK study among facilities documenting PCV10 aliquot number (data not shown).

**Table 1:** Number of pneumococcal conjugate vaccine (PCV10) and pentavalent vaccine immunizations administered to infants in VAEIK study sites according to dose number, Kenya, 14 February 2011–13 February 2013.

Study site and dose number^a^	No. of Immunizations					
	14 Feb 2011–13 Feb 2012		14 Feb 2012–13 Feb 2013		Total	
	PCV	Pentavalent Vaccine	PCV	Pentavalent Vaccine	PCV	Pentavalent Vaccine
**Kilifi HDSS** ^**b**^						
Dose 1	12,846	7,625	5,857	5,918	18,703	13,543
Dose 2	11,292	7,728	5,595	5,651	16,887	13,379
Dose 3	9,871	7,428	5,537	5,730	15,408	13,158
Total	34,009	22,781	16,989	17,299	50,998	40,080
**Kibera PBIDS**						
Dose 1	734	509	356	350	1,090	859
Dose 2	592	408	283	285	875	693
Dose 3	478	369	291	290	769	659
Unknown dose	6	2	6	7	12	9
Total	1,810	1,288	936	932	2,746	2,220
**Rarieda PBIDS**						
Dose 1	548	443	425	433	973	876
Dose 2	539	401	392	399	931	800
Dose 3	389	343	370	372	759	715
Total	1,476	1,187	1,187	1,204	2,663	2,391
**KEMRI/CDC HDSS**						
Dose 1	400	286	263	256	663	542
Dose 2	391	273	253	252	644	525
Dose 3	315	249	259	258	574	507
Total	1,106	808	775	766	1,881	1,574
**All study sites**						
Dose 1	14,528	8,863	6,901	6,957	21,429	15,820
Dose 2	12,814	8,810	6,523	6,587	19,337	15,397
Dose 3	11,053	8,389	6,457	6,650	17,510	15,039
Unknown dose	6	2	6	7	12	9
Total	38,401	26,064	19,887	20,201	58,288	46,265

Abbreviations: CDC, Centers for Disease Control and Prevention; HDSS, Health and Demographic Surveillance System; PBIDS, Population-based Infectious Disease Surveillance Site; KEMRI, Kenya Medical Research Institute; VAEIK, Vaccine Adverse Events in Kenya

^a^“Dose number” refers to the ordinal number of a vaccination within the 3-dose series for PCV or pentavalent vaccine—the first vaccination of a given child with PCV was termed “dose 1,” the second vaccination of the same child with PCV was termed “dose 2,” and the third vaccination of the same child with PCV was termed “dose 3” (likewise for pentavalent vaccine).

^b^Kilifi HDSS data include 5,232 doses of PCV (4,971 dose 1; 261 dose 2) and 2,118 doses of pentavalent vaccine (628 dose 1; 801 dose 2; 689 dose 3) administered from 11 Jan 2011–13 Feb 2011.

**Table 2:** Number of pneumococcal conjugate vaccine (PCV10) immunizations administered to infants in VAEIK study sites according to aliquot number, Kenya, 14 February 2011–13 February 2013.

Study site and aliquot number^a^	No. of Immunizations		
	14 Feb 2011–13 Feb 2012	14 Feb 2012–13 Feb 2013	Total
**Kilifi HDSS** ^**b**^			
Aliquot 1	12,254	9,254	21,508
Aliquot 2	10,117	7,663	17,780
Unknown aliquot	11,638	72	11,710
*% Unknown*	34.2%	0.42%	23.0%
Total	34,009	16,989	50,998
**Kibera PBIDS** ^**c**^			
Aliquot 1	342	170	512
Aliquot 2	256	104	360
Unknown aliquot	1,212	662	1,874
*% Unknown*	*67*.*0%*	*70*.*7%*	*68*.*2%*
Total	1,810	936	2,746
**Rarieda PBIDS** ^**c**^			
Aliquot 1	478	523	1,001
Aliquot 2	409	395	804
Unknown aliquot	589	269	858
*% Unknown*	*39*.*9%*	*22*.*7%*	*32*.*2%*
Total	1,476	1,187	2,663
**KEMRI/CDC HDSS**			
Aliquot 1	597	436	1,033
Aliquot 2	427	331	758
Unknown aliquot	82	8	90
*% Unknown*	*7*.*4%*	*1*.*0%*	*4*.*8%*
Total	1,106	775	1,881
**All study sites**			
Aliquot 1	13,671	10,383	24,054
Aliquot 2	11,209	8,493	19,702
Unknown aliquot	13,521	1,011	14,532
*% Unknown*	*35*.*2%*	*5*.*1%*	*24*.*9%*
Total	38,401	19,887	58,288

Abbreviations: CDC, Centers for Disease Control and Prevention; HDSS, Health and Demographic Surveillance System; PBIDS, Population-based Infectious Disease Surveillance Site; KEMRI, Kenya Medical Research Institute; VAEIK, Vaccine Adverse Events in Kenya

^a^“Aliquot number” refers to the order in which quantities of vaccine were removed from a multidose vial for administration to a child—the first quantity of vaccine removed from a given vial was termed “aliquot 1” and the second quantity removed from the same vial was termed “aliquot 2.”

^b^Kilifi HDSS data include 5,232 doses of PCV (36 aliquot 1; 6 aliquot 2; 5,190 unknown aliquot) administered from 11 Jan 2011–13 Feb 2011.

^c^Data shown are for all health facilities in each PBIDS. However, only a subset of facilities participated in recording aliquot information. Of the total doses of PCV administered in each PBIDS site, 35.8% and 86.2% were administered in aliquot recording facilities in Kibera and Rarieda, respectively. Among the facilities that recoded aliquot information, the overall percentage of unknown aliquots was 11.4% in the Kibera PBIDS site and 21.4% in the Rarieda PBIDS site.

### PCV10 Abscess Risk by Aliquot Number

Among all study sites, a total of 16 injection-site abscesses were identified within 7 days following injections of PCV10 for which aliquot number was recorded (Table 3). Overall, there was no statistically significant difference in risk of abscess following injection with the second (8 abscesses, 41 abscesses per 100,000 injections) compared to the first (8 abscesses, 33 abscesses per 100,000 injections) aliquot of PCV10 (RR 1.22, 95% confidence interval [CI] 0.37–4.06). There also were no significant differences in risks of abscess or related symptoms (PBIDS sites) according to PCV10 aliquot number in individual study sites (Table 3).

**Table 3:** Risk of developing abscess or similar symptoms within 7 days in relation to pneumococcal conjugate vaccine (PCV10) injection, according to whether the child received a first or second aliquot of PCV in VAEIK study sites, Kenya, 14 February 2011–13 February 2013.

Study site and syndrome	1^st^ Aliquot			2^nd^ Aliquot			Risk Ratio		
	n	N^a^	n/N per 100,000	n	N^a^	n/N per 100,000	2^nd^/1^st^ Aliquot	95% CI	p-value
**Kilifi HDSS** ^**b**^									
Abscess	2	21,508	9	1	17,780	6	0.60	[0.05–6.67]	0.68
**Kibera PBIDS** ^**c**^ ^**,**^ ^**d**^									
Abscess	0	344	0	0	200	0	-	-	-
Swelling	21	344	6,105	11	200	5,500	0.90	[0.46–1.78]	0.76
Discharge	1	344	291	0	200	0	0.00	-	1.00
Redness	13	344	3,779	4	200	2,000	0.53	[0.16–1.76]	0.30
**Rarieda PBIDS** ^**d**^									
Abscess	6	1,001	599	7	804	871	1.45	[0.36–5.90]	0.60
Swelling	228	1,001	22,777	189	804	23,507	1.03	[0.88–1.22]	0.71
Discharge	8	1,001	799	8	804	995	1.25	[0.36–4.31]	0.73
Redness	22	1,001	2,198	23	804	2,861	1.30	[0.72–2.35]	0.38
**KEMRI/CDC HDSS**									
Abscess	0	1,033	0	0	758	0	-	-	-
**All study sites**									
Abscess	8	23,886	33	8	19,542	41	1.22	[0.37–4.06]	0.74

Abbreviations: CDC, Centers for Disease Control and Prevention; CI, confidence interval; HDSS, Health and Demographic Surveillance System; PBIDS, Population-based Infectious Disease Surveillance Site; KEMRI, Kenya Medical Research Institute; VAEIK, Vaccine Adverse Events in Kenya

^a^Denominator is the number of vaccine doses administered.

^b^Denominators for Kilifi HDSS include 36 1^st^ aliquots and 6 2^nd^ aliquots administered from 11 Jan 2011–13 Feb 2011; no abscess events following PCV injection occurred during this time period.

^c^Excludes observations for the period from February 2011 through June 2011 because it was not documented whether syndromes were related to PCV injection sites or to pentavalent vaccine injection sites during this period.

^d^PBIDS data represent household- and health facility-based surveillance combined; no abscesses or related symptoms were identified at the PBIDS health facilities.

### Abscess Risk of PCV10 Compared to Pentavalent Vaccines

Among a total of 38,958 matched vaccination encounters in which the infant received both PCV10 and pentavalent vaccine, 17 injection site abscesses were identified within 7 days following PCV10 injection (44/100,000 injections) and 43 abscesses were identified within 7 days following pentavalent vaccine injection (110/100,000 injections; matched odds ratio [mOR] 0.30, 95% CI 0.16–0.58; p = 0.0002) (Table 4). Across sites, PCV10-related injection site abscesses ranged from zero in the KEMRI/CDC HDSS site and the Kibera PBIDS site to 14 in the Rarieda PBIDS site, and pentavalent vaccine-related abscesses ranged from zero in the KEMRI/CDC HDSS site to 25 in the Rarieda PBIDS site. Compared to pentavalent vaccine, PCV10 was associated with statistically significantly lower risk of injection site abscess in the Kilifi HDSS (mOR 0.17, 95% CI 0.04–0.75), of care-giver reported injection site swelling in both PBIDS sites (Kibera: mOR 0.30, 95% CI 0.20–0.46; Rarieda: mOR 0.27, 95% CI 0.22–0.32), and of caregiver-reported discharge (mOR 0.43, 95% CI 0.22–0.85) and redness (mOR 0.30, 95% CI 0.21–0.44) in the Rarieda PBIDS site.

**Table 4:** Matched analysis of the risk of developing abscess or similar symptoms within 7 days in relation to pneumococcal conjugate vaccine (PCV10) injection and pentavalent (combined GSK & SII) vaccine injection in VAEIK study sites, Kenya, 14 February– 13 February 2013.

Study site and syndrome	GSK & SII-pentavalent Vaccine			PCV					
	n	N^a^	n/N per 100,000	n	N^a^	n/N per 100,000	OR	95% CI	p-value
**Kilifi HDSS**									
Abscess	13	33,724	39	3	33,724	9	0.17	[0.04–0.75]	0.0129
**Kibera PBIDS** ^**b**^									
Abscess	5	1,577	317	0	1,577	0	0.00	-	0.0623
Swelling	158	1,577	10,019	97	1,577	6,151	0.30	[0.20–0.46]	< .0001
Discharge	5	1,577	317	5	1,577	317	1.00	[0.17–5.99]	1.0000
Redness	48	1,577	3,044	44	1,577	2,790	0.73	[0.33–1.59]	0.5483
**Rarieda PBIDS** ^**b**^									
Abscess	25	2,182	1,146	14	2,182	642	0.46	[0.21–0.98]	0.0616
Swelling	810	2,182	37,122	491	2,182	22,502	0.27	[0.22–0.32]	< .0001
Discharge	34	2,182	1,558	19	2,182	871	0.43	[0.22–0.85]	0.0201
Redness	132	2,182	6,049	57	2,182	2,612	0.30	[0.21–0.44]	< .0001
**KEMRI/CDC HDSS**									
Abscess	0	1,475	-	0	1,475	0	-	-	-
**All study sites**									
Abscess	43	38,958	110	17	38,958	44	0.30	[0.16–0.58]	0.0002

Abbreviations: CDC, Centers for Disease Control and Prevention; CI, confidence interval; GSK, GlaxoSmithKline; HDSS, Health and Demographic Surveillance System; PBIDS, Population-based Infectious Disease Surveillance Site; KEMRI, Kenya Medical Research Institute; OR, odds ratio; SII, Serum Institute of India; VAEIK, Vaccine Adverse Events in Kenya

^a^Denominator is the number of vaccine doses administered. Data represent matched vaccine encounters in which the infant received both pentavalent vaccine and PCV10 at the same visit.

^b^PBIDS data represent household- and health facility-based surveillance combined; no abscesses or related symptoms were identified at the PBIDS health facilities.

The risks of abscess per 100,000 injections for the SII versus GSK pentavalent vaccines were, respectively, 44 and 0 in the Kilifi HDSS site (RR ∞, p = 0.088), 1,304 and 363 in the Rarieda PBIDS site (RR 3.55, 95% CI 0.47–26.92, p = 0.22), and 108 and 22 across all study sites except the Kibera PBIDS site (RR 4.84, 95% CI 0.66–35.64, p = 0.12) (Table 5). In matched analyses stratified by pentavalent vaccine formulation, risk of abscess did not differ significantly between PCV10 and GSK pentavalent vaccine in the two HDSS sites (risk zero per 100,000 injections for both vaccines in both sites) or the Rarieda PBIDS site (risk 475 and 475 per 100,000 injections for PCV10 and GSK pentavalent vaccine, respectively; mOR 1.00, 95% CI 0.12–8.56) (Table 6). For data combined across all sites, abscess risk was significantly lower for PCV10 (47 abscesses per 100,000 injections) than for SII pentavalent vaccine (128 abscesses per 100,000 injections; mOR 0.27, 95% CI 0.14–0.54) (Table 7). Caregiver-reported injection site swelling was significantly less common following immunization with PCV10 than with either pentavalent vaccine formulation (Tables 6 and 7).

**Table 5:** Risk of developing abscess or similar symptoms within 7 days in relation to SII-pentavalent vaccine injection or GSK-pentavalent vaccine injection in VAEIK study sites, Kenya, 14 February 2011–13 February 2013.

Study site and syndrome	GSK-pentavalent Vaccine (2-dose vial)			SII-pentavalent Vaccine (10-dose vial)			Risk Ratio	95% CI	p-value
	n	N^a^	n/N per 100,000	n	N^a^	n/N per 100,000			
**Kilifi HDSS**									
Abscess	0	8,045^b^	0	14	32,035	44	∞	-	0.088
**Rarieda PBIDS** ^**c**^									
Abscess	2	551	363	24	1,840	1,304	3.55	[0.47–26.92]	0.22
Swelling	191	551	34,664	680	1,840	36,957	1.07	[0.91–1.25]	0.43
Discharge	4	551	726	32	1,840	1,739	2.38	[0.56–10.19]	0.24
Redness	37	551	6,715	109	1,840	5,924	0.88	[0.57–1.37]	0.58
**KEMRI/CDC HDSS**									
Abscess	0	350	0	0	1224	0	-	-	-
**All sites**									
Abscess	2	8,946	22	38	35,099	108	4.84	[0.66–35.64]	0.12

Abbreviations: CDC, Centers for Disease Control and Prevention; CI, confidence interval; GSK, GlaxoSmithKline; HDSS, Health and Demographic Surveillance System; PBIDS, Population-based Infectious Disease Surveillance Site; KEMRI, Kenya Medical Research Institute; SII, Serum Institute of India; VAEIK, Vaccine Adverse Events in Kenya

^a^Denominator is the number of vaccine doses administered.

^b^Includes 2,118 doses of GSK-pentavalent vaccine administered from 11 Jan 2011–13 Feb 2011.

^c^PBIDS data represent household- and health facility-based surveillance combined; no abscesses or related symptoms were identified at the PBIDS health facility.

**Table 6:** Matched analysis of the risk of developing abscess or similar symptoms within 7 days in relation to pneumococcal conjugate vaccine (PCV10) injection and GSK-pentavalent vaccine injection in VAEIK study sites, Kenya, 14 February 2011–13 February 2013.

Study site and syndrome	GSK-pentavalent Vaccine			PCV					
	n	N^a^	n/N per 100,000	n	N^a^	n/N per 100,000	OR	95% CI	p-value
**Kilifi HDSS** ^**b**^									
Abscess	0	6,271	0	0	6,271	0	-		-
**Rarieda PBIDS** ^**c**^									
Abscess	2	421	475	2	421	475	1.00	[0.12–8.56]	1.0000
Swelling	160	421	38,005	123	421	29,216	0.49	[0.33–0.73]	0.0004
Discharge	3	421	713	3	421	713	1.00	[0.16–6.14]	1.0000
Redness	29	421	6,888	17	421	4,038	0.49	[0.24–0.98]	0.0597
**KEMRI/CDC HDSS**									
Abscess	0	267	-	0	267	0	-		
**All study sites**									
Abscess	2	6,959	29	2	6,959	29	1.00	[0.12–8.56]	1.000

Abbreviations: CDC, Centers for Disease Control and Prevention; CI, confidence interval; GSK, GlaxoSmithKline; HDSS, Health and Demographic Surveillance System; PBIDS, Population-based Infectious Disease Surveillance Site; KEMRI, Kenya Medical Research Institute; OR, odds ratio; VAEIK, Vaccine Adverse Events in Kenya

^a^Denominator is the number of vaccine doses administered. Data represent matched vaccine encounters in which the infant received both pentavalent vaccine and PCV10 at the same visit.

^b^Denominators for Kilifi HDSS include 1,719 matched doses of PCV and GSK-pentavalent vaccine administered from 11 Jan 2011–13 Feb 2011.

^c^PBIDS data represent household- and health facility-based surveillance combined; no abscesses or related symptoms were identified at the PBIDS health facility.

**Table 7:** Matched analysis of the risk of developing abscess or similar symptoms within 7 days in relation to pneumococcal conjugate vaccine (PCV10) injection and SII-pentavalent vaccine injection in VAEIK study sites, Kenya, 14 February 2011–13 February 2013.

Study site and syndrome	SII-pentavalent Vaccine			PCV					
	n	N^a^	n/N per 100,000	n	N^a^	n/N per 100,000	OR	95% CI	p-value
**Kilifi HDSS**									
Abscess	13	27,453	47	3	27,453	11	0.17	[0.04–0.75]	0.0129
**Kibera PBIDS** ^**b**^									
Abscess	5	1,577	317	0	1,577	0	0.00	-	0.0623
Swelling	158	1,577	10,019	97	1,577	6,151	0.30	[0.20–0.46]	< .0001
Discharge	5	1,577	317	5	1,577	317	1.00	[0.17–5.99]	1.0000
Redness	48	1,577	3,044	44	1,577	2,790	0.73	[0.33–1.59]	0.5483
**Rarieda PBIDS** ^**b**^									
Abscess	23	1,761	1,306	12	1,761	681	0.41	[0.18–0.93]	0.0465
Swelling	650	1,761	36,911	368	1,761	20,897	0.22	[0.18–0.27]	< .0001
Discharge	31	1,761	1,760	16	1,761	909	0.37	[0.18–0.79]	0.0123
Redness	103	1,761	5,849	40	1,761	2,271	0.25	[0.16–0.40]	< .0001
**KEMRI/CDC HDSS**									
Abscess	0	1,208	-	0	1,208	0	-	-	-
**All study sites**									
Abscess	41	31,999	128	15	31,999	47	0.27	[0.14–0.54]	0.0001

Abbreviations: CDC, Centers for Disease Control and Prevention; CI, confidence interval; HDSS, Health and Demographic Surveillance System; PBIDS, Population-based Infectious Disease Surveillance Site; KEMRI, Kenya Medical Research Institute; OR, odds ratio; SII, Serum Institute of India; VAEIK, Vaccine Adverse Events in Kenya

^a^Denominator is the number of vaccine doses administered. Data represent matched vaccine encounters in which the infant received both pentavalent vaccine and PCV10 at the same visit.

^b^PBIDS data represent household- and health facility-based surveillance combined; no abscesses or related symptoms were identified at the PBIDS health facilities.

## Discussion

In this 2-year, multisite Phase IV study, we did not find evidence of increased risk of injection site abscess following infant immunization with the second compared to the first aliquot of a 2-aliquot, preservative-free formulation of 10-valent pneumococcal conjugate vaccine introduced into Kenya’s routine immunization program in 2011. Further, no increased risk of abscesses was seen following PCV10 compared to pentavalent vaccine (either formulation) administered according to the same 3-dose schedule as PCV10. To our knowledge, this was the first and to-date is the longest Phase IV study of a multi-aliquot, preservative-free vaccine formulation used routinely in a developing country, and our findings suggest that 2-aliquot vial formulations of such vaccines have the potential for safe use in developing country settings, assuming adherence to policies regarding storage and use. We made the incidental observation that the risk of abscess following the 10-dose formulation of pentavalent vaccine was higher than the risk following the 2-dose formulation. This difference was large but it was not statistically significant. Our finding that the 2-aliquot, preservative-free formulation of PCV10 has been safely introduced in Kenya is important because it supports introduction of this vaccine in other developing countries where pneumococcal immunization may substantially reduce morbidity and mortality from pneumococcal disease among young children [10–11].

For our primary analysis, evaluating the abscess risk of second compared to first aliquot injections of PCV10, the study had sufficient power to detect only large effect sizes. We calculate that all study sites combined had 86% power to detect a risk ratio of 3.5 but only 56% power to detect a risk ratio of 2.5 for abscesses associated with aliquot 2 when compared with aliquot 1. Smaller differences in abscess risk between PCV10 aliquots 1 and 2 might not have been detected because of insufficient sample size. To detect a 2-fold increased risk—which would be of considerable public health importance—the study would need to have been 2.4 times larger overall. Encouragingly, a one-year study of PCV10 AEFI in Ethiopia that was patterned on VAEIK methods also found no statistically significant increased risk of injection site abscess following immunization with the second compared to the first aliquot of PCV10 [12].

The comparison of abscess and related symptom risks of PCV10 and pentavalent vaccine was complicated by the change in the formulation of pentavalent vaccine used in Kenya’s routine immunization program during the study period, from a 2-aliquot presentation manufactured by GSK to a 10-aliquot presentation manufactured by the Serum Institute of India. Because of the potential difference in abscess risks related to the two pentavalent vaccine formulations, we performed stratified analyses comparing PCV10 abscess risk separately to the GSK and SII pentavalent vaccine products, in addition to the planned comparison of abscess risk between PCV10 and pentavalent vaccine over the entire 2-year study period. None of these analyses showed increased risk of abscess following immunization with PCV10 compared to the pentavalent vaccine products, providing reassurance that the preservative-free 2-aliquot vial formulation of PCV10 may be used safely in Kenya. Nevertheless, the power of the study to detect differences in vaccine safety between PCV10 and the originally intended comparator, the GSK pentavalent vaccine, was reduced in the stratified analysis.

Given the heterogeneity in our surveillance methods (active versus passive surveillance), differences in risk estimates across sites, and a lack of external validation of our abscess case definitions, a concern might exist that our failure to find an increased abscess risk for the second compared to first aliquot of PCV10 could reflect bias toward the null resulting from nondifferential misclassification errors related to exposures and/or outcomes. We do not have information on the frequency of potential misclassification of the first versus second aliquot of PCV10, which could have reduced the observed effect sizes. However, the incidental observation of an increased abscess risk of the SII compared to GSK pentavalent vaccine confirms the potential of the surveillance methodology to detect systematic differences in abscess risk for products within Kenya’s routine immunization program. Thus, as the VAEIK study did have the capacity to identify programmatic associations between specific vaccine exposures and risks of injection site abscess, it is likely that the study would have identified similar excess risk related to the second aliquot of PCV10, if one had existed.

Our estimates of abscess risk should be interpreted in light of the different surveillance practices used across sites. Active surveillance, which was used only in the two PBIDS sites, is normally associated with higher ascertainment of AEFIs, especially for mild symptoms. This is likely to account for our findings that the estimates of abscess risk following immunization were 10–100 times greater in the PBIDS sites compared to the Kilifi HDSS site. Despite standard training in the recognition and case-definition of abscess at each study site, inherent differences between active and passive surveillance processes raise the possibility that the clinical entities classified as abscess may also have differed across sites.

We incidentally observed that the risk of abscess was 4.8-fold higher across study sites following immunization with the 10-aliquot vial SII pentavalent vaccine formulation compared to the 2-aliquot vial GSK pentavalent vaccine formulation. This association was not statistically significant and could have resulted from sampling error. The association most closely approached statistical significance in the Kilifi HDSS site (p = 0.088), which contributed the largest share of vaccine observations among the four study sites. It also is possible that some proportion of the observed difference in abscess risk for the SII versus GSK pentavalent vaccine could have resulted from increased sensitivity of VAEIK surveillance procedures over time, or from other aspects of the VAEIK study design that were not tailored to properly evaluate the safety of the change in pentavalent vaccine formulation. There may have been misclassification of exposure regarding the formulation of pentavalent vaccine used during the period of transition from GSK to SII pentavalent vaccine in Kenya’s routine immunization program, although such misclassification would have biased our comparison of abscess risk between the pentavalent vaccine formulations toward the null. If the observed association is not attributable to sampling error or ascertainment bias, then it would have significant implications for vaccine policy in Kenya and elsewhere in the developing world. The risk difference between pentavalent vaccine formulations is most interpretable in the Rarieda PBIDS site, where non-zero abscess incidences were observed for both pentavalent vaccine products—there, the risk difference between the 10-aliquot vial and the 2-aliquot vial pentavalent vaccine formulations was roughly 1% (1.30% risk for 10-aliquot vial pentavalent vaccine minus 0.36% risk for 2-aliquot vial pentavalent vaccine), suggesting that 1% of children receiving the 10-aliquot vial pentavalent vaccine may develop abscesses attributable to the new formulation. Abscesses in the Rarieda PBIDS site were identified through active household surveillance which, as noted above, could identify abscess events not severe enough to prompt a health facility visit. Nevertheless, Kenya administers approximately 5 million doses of pentavalent vaccine per year, which, with the 10-aliquot vial product, could be responsible for 50,000 unnecessary abscesses, some proportion of which may be associated with more severe clinical manifestations, like bacteremia and sepsis. The magnitude of the risk ratio across two sites suggests that the potential of contamination of doses of the 10-aliquot vial pentavalent vaccine in Kenya should be further evaluated and additional training on proper handling and administration of this pentavalent vaccine should be considered. Inclusion of a preservative may not wholly counter the risk of contamination in 10-aliquot vial vaccine presentations used in developing countries.

This study provides important lessons for vaccine safety monitoring in developing countries. The infrastructure for vaccine safety surveillance is often limited in the very settings that are most in need of expanded access to immunizations. In the current study, even the combination of four field study sites in Kenya with existing surveillance capacity provided a study power of only ~80% to detect a three-fold increased risk of injection site abscess, which is a relatively common potential AEFI. Less frequent but more serious AEFI would be even more difficult to detect with existing surveillance systems. As new vaccines are introduced into GAVI countries, the potential for undetected adverse events is considerable. In addition, the capacity of national immunization programs and global vaccine policy bodies to refute spurious associations (e.g., autism and vaccines) is determined by the robustness of AEFI surveillance systems. HDSS sites and similar health surveillance platforms in developing countries provide useful frameworks for AEFI surveillance, but more such platforms are needed and specific effort and resources should be allocated to develop effective AEFI surveillance programs in multiple sites. The potential for breaks in sterile technique and vaccine-handling practices likely differs from site to site and in different countries in Africa, so substantial additional experience and more post-licensure observational studies will be needed to fully characterize risks associated with use of preservative-free, multi-dose vial vaccine formulations such as PCV10 in developing countries.

## Conclusions

In summary, this Phase IV multisite study found no evidence to suggest that use of a 2-aliquot vial, preservative-free formulation of PCV10 in Kenya’s routine immunization program was associated with an increased risk of injection site abscesses, related to the second aliquot or overall. The incidental observation of an increased risk of abscess associated with a change in the formulation of the comparator vaccine (pentavalent) during the study period, while complicated, confirms the capacity of the VAEIK surveillance methods to detect relevant, clinically important changes in the safety profile of vaccines administered in Kenya’s routine immunization program and provides context for the findings regarding PCV10.
